# CAPS: a simple clinical tool for β-amyloid positivity prediction in clinical Alzheimer syndrome

**DOI:** 10.3389/fneur.2024.1422681

**Published:** 2024-08-14

**Authors:** Durjoy Lahiri, Bruna Seixas-Lima, Carlos Roncero, Nicolaas Paul Verhoeff, Morris Freedman, Sarmad Al-Shamaa, Howard Chertkow

**Affiliations:** ^1^Baycrest Academy for Research and Education/Rotman Research Institute, University of Toronto, Toronto, ON, Canada; ^2^Department of Neurology, Institute of Neurosciences Kolkata, Kolkata, India

**Keywords:** Alzheimer, clinical, CAPS, early diagnosis, amyloid-beta, predictivity

## Abstract

**Introduction:**

With the advent of anti-β-amyloid therapies, clinical distinction between Aβ + and Aβ− in cognitive impairment is becoming increasingly important for stratifying referral and better utilization of biomarker assays.

**Methods:**

Cognitive profile, rate of decline, neuropsychiatric inventory questionnaire (NPI-Q), and imaging characteristics were collected from 52 subjects with possible/probable AD.

**Results:**

Participants with Aβ+ status had lower baseline MMSE scores (24.50 vs. 26.85, *p* = 0.009) and higher total NPI-Q scores (2.73 vs. 1.18, *p* < 0.001). NPI-Q score was found to be the only independent predictor for β-amyloid positivity (*p* = 0.008). A simple scoring system, namely Clinical β-Amyloid Positivity Prediction Score (CAPS), was developed by using the following parameters: NPI-Q, rapidity of cognitive decline, and white matter microangiopathy. Data from 48 participants were included in the analysis of accuracy of CAPS. CAP Score of 3 or 4 successfully classified Aβ + individuals in 86.7% cases.

**Discussion:**

Clinical β-Amyloid Positivity Prediction Score is a simple clinical tool for use in primary care and memory clinic settings to predict β-amyloid positivity in individuals with clinical Alzheimer Syndrome can potentially facilitate referral for Anti Aβ therapies.

## 1 Introduction

One of the crucial current challenges in the care of dementia patients revolves around the observation that many individuals who clinically meet criteria for probable or possible Alzheimer Disease (AD), turn out on more detailed biochemical assessment to be amyloid negative—they have normal amyloid on cerebrospinal fluid (CSF) testing and are negative on β-amyloid positron emission tomography (PET). Serrano-Pozo et al. (1) found that 14% of a large cohort of participants with a clinical diagnosis of mild to moderate AD had no or sparse β-amyloid plaques in their brains. Monsell et al. (2) reported that 1/3rd of non-Apo E4 carrier patients clinically diagnosed with mild to moderate AD did not have amyloid in their brain post-mortem. In the Imaging Dementia-Evidence for Amyloid Scanning (IDEAS) study, where β-amyloid PET was offered to a large group of AD patients (3), the β-amyloid negative figure was closer to 1/3 of referred patients. These individuals might be best described as Alzheimer Syndrome. Several explanations have been proposed to account for this clinical-biological gap (4–8), including disease entities such as hippocampal sclerosis (9), limbic predominant age-related TDP-43 encephalopathy (10), primary age-related tauopathy (11). Yet there is no good predictor readily available to distinguish between the group of individuals with clinical “Alzheimer Syndrome” versus those with pathological AD. This can be ascribed to the lack of studies investigating the clinical difference between these sub-groups. For instance, what demographic, clinical or imaging factors are more frequent in the β-amyloid positive (Aβ+) group as compared to their β-amyloid negative (Aβ−) counterparts?

Clinically distinguishing factors between Aβ + and Aβ− subgroups with cognitive impairment reported in the literature are limited to longitudinal cognitive performance, use of antidepressants, and baseline Mini Mental Status Examination (MMSE) score (12). However, biomarker differences between these subgroups are reportedly more extensive. For instance, Aβ− people tend to have different atrophy patterns on magnetic resonance imaging (MRI) and [^18^F]flurodeoxyglucose (FDG) PET metabolism in certain brain regions than their Aβ + counterparts (13). Phospho-tau and total-tau have been found to be more elevated in Aβ + people than the Aβ− subgroup (14). White matter hyperintensities and cerebral microbleeds are also documented to be more frequent in Aβ + people (15). While biomarker differences are important, these features are not immediately available to primary care and even specialty physicians. It is an open question as to whether there are key clinical features, which might lead a clinician to suspect that an individual is Aβ− despite receiving the clinical diagnosis of AD. With the advent of anti-β-amyloid therapies, clinical distinction between these two subgroups is becoming increasingly important for stratifying referral for these treatments, along with better utilization of biomarker assays in people living with AD.

The objective of this study was to address the question as to whether there are clinical features available which can help to distinguish Aβ + from Aβ− subjects. If so, such features might be used to develop a prediction model for clinicians dealing with AD. Ideally, this tool should be able to inform clinical decision making for β-amyloid biomarker analysis. This will not only help in judicious use of available resources but also will help physicians in properly advising people with Alzheimer Syndrome, who might be candidates to receive anti-β-amyloid therapy.

## 2 Methods

This is a retrospective observational study conducted in an academic memory clinic located in Toronto, Ontario, Canada. Prior ethical approval was obtained from the Research Ethics Board. Informed written consent was collected from each participant or their substitute decision maker when deemed appropriate due to the participant’s limited cognitive capacity.

A retrospective chart review of 52 probable AD/prodromal AD patients screened for anti-β-amyloid clinical trials was performed. Either for clinical assessment reasons, or for screening purposes for clinical trials, evaluation of β-amyloid status was carried out either via CSF or PET β-amyloid studies. For purposes of this study, our focus was to subdivide subjects who were Aβ + versus Aβ−.

The following information was collected from the chart review—demographic information, medical history, clinical presentation, course of disease and rate of progression, neuropsychological test scores, blood work, brain scans, and any other relevant investigations.

### 2.1 Cognitive assessment

Mini Mental Status Examination (16) and Montreal Cognitive Assessment (MoCA) tests (17) were administered as standard cognitive assessments during their evaluations. Wherever MoCA was available only, MMSE equivalence was calculated using a standard conversion table (18). While a range of MMSE scores are possible for any given MoCA score, the weighted mean scores provided in the conversion were accepted only for the current study. Table 1 represents the subjects whose MoCA scores were converted to equivalent MMSE scores. It has been documented in literature that conversion of MoCA to MMSE is more accurate than the reverse. Only MMSE scores were considered in the final analysis as a measure of cognitive status.

**Table 1:** MoCA and equivalent MMSE scores of select subjects for whom MMSE was not available.

Subject ID	MoCA	Corrected MMSE scores using weighted mean
014	15	21
023	28	29
024	11	18
001	25	28
003	12	19
005	30	30
006	26	29
002	21	26
026	26	29
027	24	28
029	13	20
034	21	26
036	14	20
039	18	24

Lawton Brody scale (19) was applied for assessment of independence in performing basic and instrumental activities of daily living (ADL) by an individual. Anyone with a score of 23/23 was denoted as fully independent for ADLs and would eventually be clinically classified as mild cognitive impairment or prodromal AD, while any documented restriction of ADLs was used as a classifier favoring clinical dementia stage of AD.

### 2.2 Rate of decline

Rate of decline was calculated by using the longitudinal MMSE scores. The difference in the MMSE scores across time points was divided by the intervening duration of time. Any decline of more than 2 points/year was considered as rapid decline for this study. Individuals (*n* = 4) without an estimate of cognitive scores at least at two time points were excluded from the final analysis wherever rapid cognitive decline was employed as a variable.

### 2.3 Neuropsychiatric inventory questionnaire

The Neuropsychiatric Inventory Questionnaire (NPI-Q) is a questionnaire-based assessment of neuropsychiatric symptoms (NPS) in dementia (20) that has 12 components along with a severity score (0–3) for each of these components. These components are delusions, hallucinations, agitation, apathy, anxiety, depression, euphoria, disinhibition, irritability, aberrant motor behavior, sleeping disorder, and appetite disturbance. The total number of NPS can be calculated from this scoring system as well as the NPI score, which is a combined reflection of the frequency and severity of NPS in dementia.

### 2.4 Hachinski score

The Hachinski Ischemic Score is a simple clinical tool used to differentiate between primary and vascular dementia (21). It considers several demographic and clinical factors to classify cognitive decline into primarily degenerative and vascular etiology. A score of more than 4 is suggestive of vascular dementia while a score of 4 or less favors non-vascular cognitive impairment.

### 2.5 Magnetic resonance imaging

Magnetic resonance imaging was carried out using 1.5 T scanners. Sections of 0.5 mm were available through these clinical MR images. T1W, T2W, and FLAIR sequences were carried out within the axial, coronal, and sagittal sections. The Scheltens score for medial temporal atrophy was calculated in T2W coronal sections of the brain images (22). The Fazekas score for white matter microangiopathy in the brain was calculated by using axial T2W sections (23).

### 2.6 Cerebrospinal fluid testing

Cerebrospinal fluid was collected by lumbar puncture following usual protocol at the memory clinic or clinical trials unit. The samples were collected in polypropylene tubes. CSF Aβ 42, phospho-tau, and total tau were analyzed by chemiluminescence immunoassays (Roche elecsys, second generation) and Aβ 42/40 ratios were calculated using mass spectrometry.

Cut-offs for the absolute values of biomarkers were as follows: Aβ 42 > 1,030 ng/L; phospho-tau <28 ng/L; total tau <301 ng/L. Biomarker ratio cut offs were: phospho-tau/β 42 < 0.024; Total tau/β 42 < 0.29.

### 2.7 β-amyloid PET

β-Amyloid PET scan results were available from participants in the GRADUATE study of gantenerumab (24) after the trial was closed or the participants had completed the trial. PET scans were carried out using 370 MBq of intravenous [^18^F]Florbetapir. The standardized uptake value ratio (SUVR) and centiloid values were subsequently calculated as per the GRADUATE study protocol.

### 2.8 Statistical analysis

Statistical analysis was conducted by using the Statistical Program for the Social Sciences (SPSS) software (version 29.0.2.0). For continuous variables, means between two groups were compared using independent samples *t*-test. To compare categorical variables, chi-square test was employed. Non-parametric tests of significance were used when the dataset did not pass the test of normality based on the Kolmogorov Smirnov test. Binary logistic regression analysis was used to predict the β-amyloid positivity in the study sample. Receiver Operating Characteristics (ROC) analysis was used along with calculating the *c*-statistic for all the variables.

## 3 Results

Data were collected by retrospective chart review (*n* = 52). Among them, 44.2% (*n* = 23) were clinically classified as prodromal AD and the remaining were mild AD. Within the study cohort, 30 were tested for CSF β-amyloid while 24 were tested by β-amyloid PET scanning. Thirty were identified as (Aβ+) and 22 were (Aβ−). Two participants had both CSF biomarker and β-amyloid PET scans available.

Table 2 depicts the demographic, clinical and imaging information along with the relevant *p* values in the two subgroups.

**Table 2:** Comparison of different variables between the Aβ+ and Aβ− subgroup.

Parameters	Amyloid-β positive (*n* = 30)	Amyloid-β negative (*n* = 22)	*p* values
Age (SD)	69.40 (10.64)	69.73 (10.76)	0.46
Sex (Female %)	63	45	0.20
Duration of illness @ presentation (SD)	2.87 (1.78)	3.29 (2.54)	0.32
Cognitive profile (amnestic presentation %)	90	86	0.46
Mean baseline MMSE (SD)	24.50 (2.52)	26.85 (3.71)	0.009^*^
^#^Rate of decline (slow/rapid)%	(39/61) (*n* = 28)	(50/50) (*n* = 20)	0.33
NPI score (SD)	2.73 (1.66)	1.18 (1.14)	<0.001^*^
Hachinski (SD)	2.37 (0.85)	2.45 (1.68)	0.81
White matter disease (Yes/No)%	0/90	18/82	0.052
MRI (MTLA score 0/1/2/3%)	10/60/12/4	35/40/15/0	0.31

^*^indicate statistical significance. ^#^ indicates exclusion of four cases from the tabulation.

Preliminary results of this study has been presented elsewhere (25). No statistically significant difference was found between the two subgroups in terms of age, sex, duration of illness, Hachinski score, and MRI atrophy scores. Although not statistically significant, higher occurrence of β-amyloid positivity was noted in females (*p* = 0.20); individuals with rapid decline (*n* = 48, *p* = 0.33) and less white matter disease (*p* = 0.052). Significant differences were found in the baseline MMSE and NPI-Q scores. People with Aβ+ status had lower baseline MMSE scores (24.50 vs. 26.85, *p* = 0.009) and higher total NPI-Q scores (2.73 vs. 1.18, *p* < 0.001). The mean number (1.97 vs. 0.95, *p* < 0.001) of NPS was also higher in the Aβ+ group than the Aβ− group. People with Aβ+ status had a significantly higher mean score for depression (0.80 vs. 0.14, *p* = 0.001) and irritability (0.63 vs. 0.18, *p* = 0.025) in contrast to their negative counterparts.

Logistic Regression analysis was carried out, retaining β-amyloid positivity as the binary outcome. All the factors which showed significance in univariate analysis were entered into the regression model (i.e., baseline MMSE and total NPI-Q score). Given the higher occurrence of β-amyloid positivity described earlier, we also investigated differences in sex, cognitive decline, white matter disease, and medial temporal lobe atrophy score. The NPI-Q score was found to be the only independent predictor for β-amyloid positivity (*p* = 0.008).

### 3.1 Clinical β-amyloid positivity prediction score

A Clinical β-Amyloid Positivity Prediction Score (CAPS) was developed by using the following parameters: NPS were measured using NPI-Q score, rapidity of cognitive decline as measured by MMSE, and white matter microangiopathy as measured by Fazekas score. The scoring system is described in Table 3.

**Table 3:** Variables in the CAPS scoring system and the points assigned.

CAPS variables	Status	Score received
NPI-Q	0	0
	1	1
	2 or more	2
Rapid cognitive decline	MMSE loss <2 per year	0
	MMSE loss >2 per year	1
Fazekas score (white matter cerebrovascular disease)	2 or more	0
	0 or 1	1

**Figure 1:**
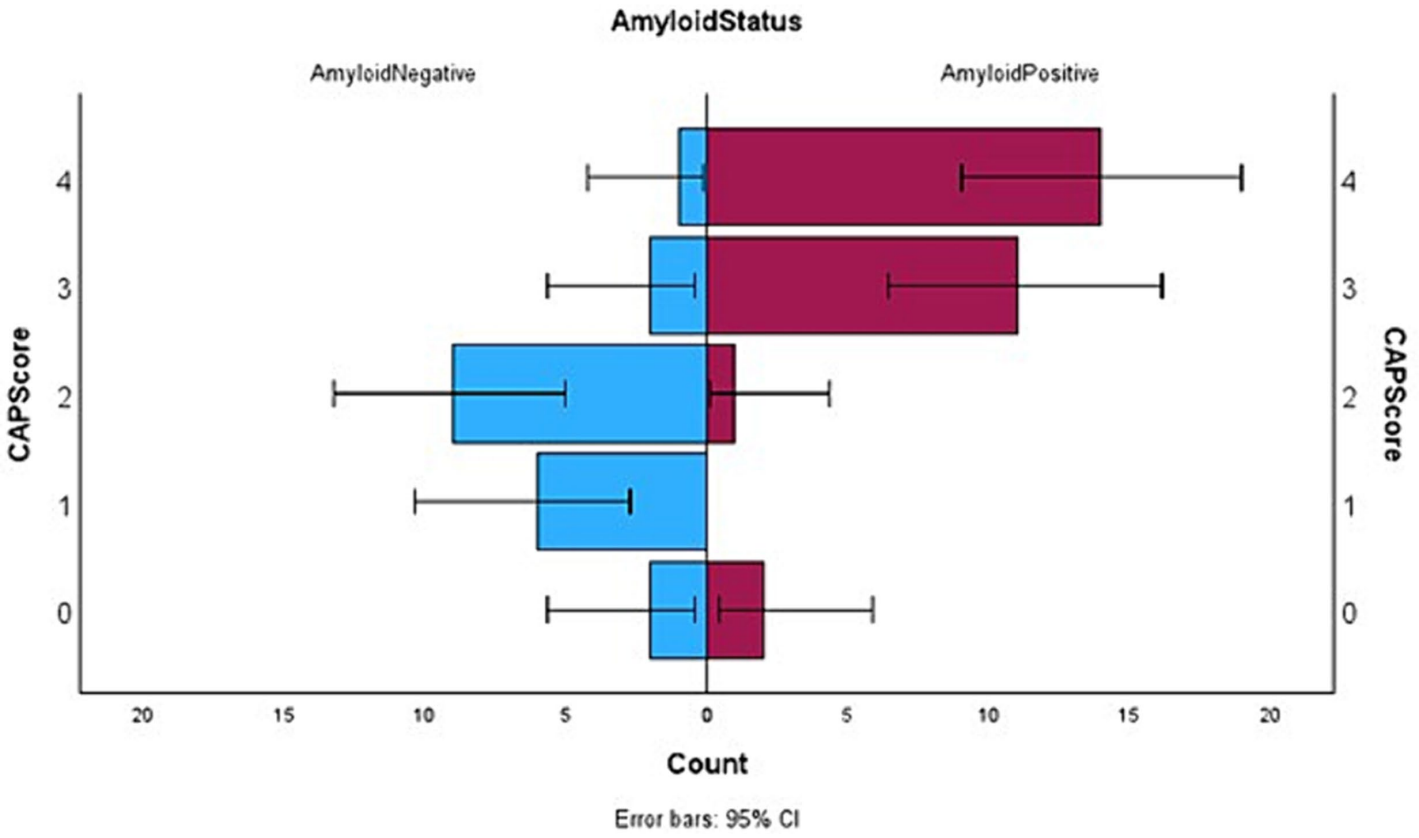
Horizontal box plots with error bars showing the distribution of CAP score between the two subgroups where Aβ positive status has been denoted on the right and Aβ negative left.

In summary, the CAPS score ranges from 0 to 4 points. The maximum score possible in CAPS is 4 (high likelihood of β-amyloid positivity) and the minimum score can be 0 (low likelihood of β-amyloid positivity). Points are assigned as 0 or 1 or 2 reflecting the total NPI-Q score obtained by an individual. Presence of neuropsychiatric symptoms, as characterized by the NPI-Q score, was found to be statistically higher in our sample in the Aβ+ group as compared to the Aβ− individuals. Therefore, the total NPI-Q score was considered as the most reliable predictor of β-amyloid positivity. Individuals with a NPS score of 2 or more received two points and a score of 1 received one point on the CAPS, while those with an NPI-Q score of 0 were assigned 0 on the CAPS. Literature (12) already has shown that people with Aβ + status decline faster than Aβ− people in terms of their cognitive scores. In accordance with this and our own results, any individual with a rapid decline (greater than 2 points on MMSE in a year) received a point of 1 in CAPS. The presence of cerebrovascular disease is a confound for the diagnosis of Alzheimer Disease. Conversely, the absence of cerebrovascular changes may imply that AD pathology is more likely to be responsible for the cognitive decline, and that β-amyloid is therefore present. Therefore, anyone with a Fazekas score of less than 2 (very little white matter microangiopathy) received a point on their CAPS score. Figure 1 depicts the difference between Aβ+ and Aβ− subgroups in terms of their CAPS.

**Figure 2:**
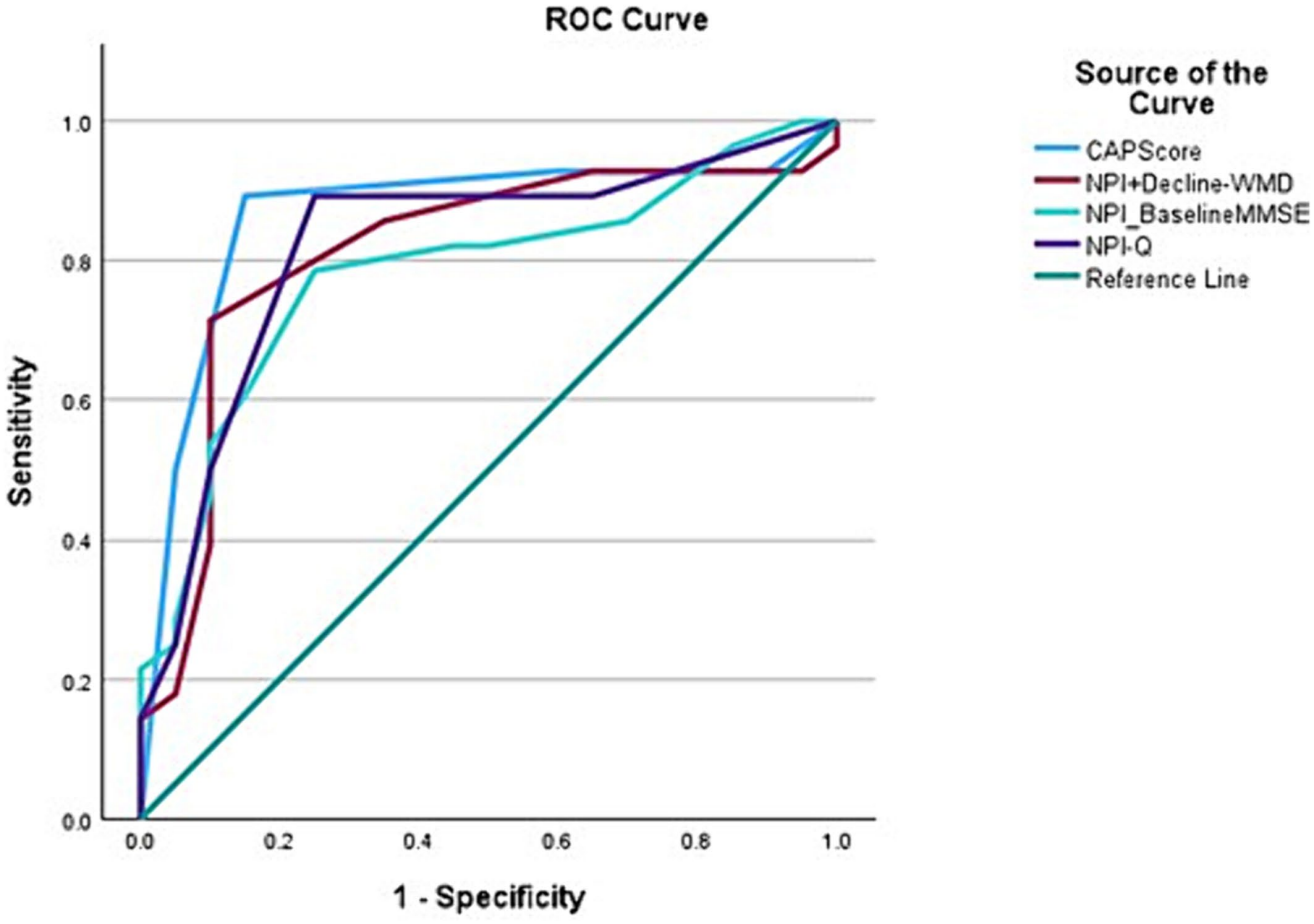
Receiver operating characteristic (ROC) curves for variables with Aβ positive status as the outcome variable demonstrating that CAPS has the best area under the curve (AUC) (0.867) when compared to other combination variables, for instance, NPI-Q+ Rapid decline-white matter disease (0.813); NPI-Q-baseline MMSE (0.783); and NPI-Q (0.821).

A total of 48 participants were included in the final analysis of accuracy of CAPS. ROC curve analysis showed that a CAPS score of 3 or 4 successfully classified Aβ+ individuals in 86.7% (95% CI, 75.2–98.2) of the cases (Figure 2). The overall model quality was found 0.75, where a good model quality value is above 0.50. In other words, 25/28 Aβ+ individuals scored 3 or 4 on CAPS while 16/20 Aβ− individuals received a score between 0 and 2. The sensitivity of CAPS therefore turns out to be 89.2% and the specificity 80%. The positive predictive value of CAPS is marginally higher than its negative predictive value (86.2 vs. 84.2%), which essentially would mean that a positive score carries more weight toward Aβ positivity (26). These figures, in general, reflect a good level of discrimination between the two subgroups under examination here. CAPS showed better discrimination than any other combination of clinical parameters, namely, NPI and baseline MMSE; NPI, rapid decline and Fazekas score alone.

**Figure 3:**
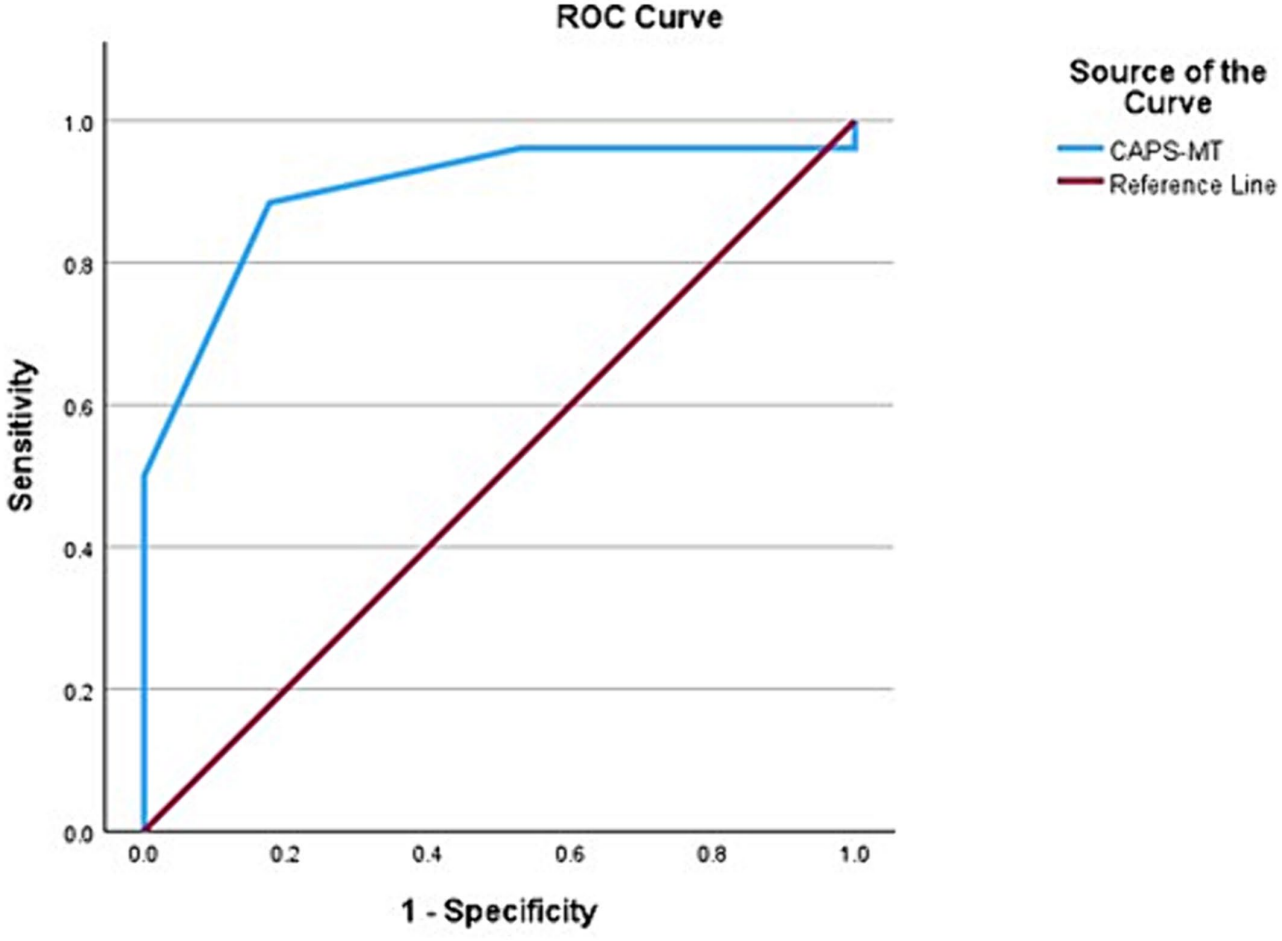
Receiver operating characteristic (ROC) curve showing the area under the curve (AUC) after medial temporal atrophy score (MTLA) was added to CAPS as an input variable. The AUC improves from 0.867 to 0.900, indicating 90% predictivity for Aβ positive status.

When CAPS was combined with an MRI biomarker for AD, namely the presence of medial temporal lobar atrophy (MTLA) as reflected in a Scheltens scale score over 2, the AUC improved to 90% (95% CI, 80.4–99.7) with an overall model quality of 0.80 (Figure 3). We propose the name CAPS-MT for when CAPS is combined with the MTLA score. The sensitivity and specificity of CAPS-MT for predicting Aβ in the brain is 92.8 and 90%, respectively, giving way to a positive likelihood ratio of 9.20 and negative likelihood ratio of 0.09.

## 4 Discussion

### 4.1 Neuropsychiatric symptoms

Neuropsychiatric symptoms (NPS) are extremely common not only in advanced AD but also in MCI and early AD (27). Contemporary research has shown that NPS are frequent in Aβ+ individuals, and they can be predictors of rapid cognitive decline to the point that agitation, irritability, and apathy are found to predict progression from MCI to dementia (28). Aβ+ individuals are more likely to develop anxiety and agitation but not depression during their cognitive decline (29). On the other hand, apathy is found more commonly in people with neurofibrillary tangles (NFTs), a downstream consequence of β-amyloid deposition in the brain (30). Mild behavioral impairment (MBI) is reportedly more common in Aβ+ individuals than their negative counterparts (31). In agreement with the previous similar studies, our study finds that the number of NPS and the frequency × severity score (which is collected in the NPI) are significantly higher in the Aβ+ subgroup than the Aβ− subgroup. Depression and irritability were the commonest NPS found in Aβ+ individuals and were significantly higher than in the Aβ− subgroup. Apathy did not show any significant differences between the two subgroups.

### 4.2 Rapid cognitive decline

Rapid cognitive decline has been found to be associated with β-amyloid positivity. For instance, Landau et al. (12) reported that Aβ+ people had lesser cognitive performance at baseline on different measures of cognition than their negative counterparts. Similarly, longitudinal cognitive performance was also worse in the Aβ+ subgroup when compared to the Aβ− individuals. Our analysis found that the baseline MMSE score was lower in the Aβ+ than the Aβ− subgroup although there was no difference between the duration of illness before presentation in the two subgroups. This would in turn indicate that the Aβ+ individuals decline at a more rapid rate than the Aβ− subgroup. A higher occurrence of rapid decline was noted among the Aβ+ subgroup which is again in agreement with existing studies. However, it should be noted that in our sample this difference did not reach statistical significance.

### 4.3 White matter disease

According to the clinical criteria for AD (32), presence of significant cerebrovascular disease or white matter disease is an important exclusion for the diagnosis of probable AD. Nevertheless, many individuals with AD have white matter changes to a variable degree. Understandably, the driving factor for cognitive decline in these individuals could be related to the vascular changes in their brain in addition to the contribution from the AD neuropathology. The relationship between β-amyloid deposition and vascular changes in the brain is bidirectional. While β-amyloid accumulation in the brain can lead to vasculopathy (33), small vessel damage can also adversely affect β-amyloid removal from the brain, promoting the development of Alzheimer senile plaque pathology (34). Likewise, studies have reported the presence of higher vascular burden in people with Aβ+ status than Aβ− (35) both radiologically (36) and pathologically (37).

Our population in this study excluded individuals suspected of having vascular or mixed dementia, since such individuals would not qualify for anti-β-amyloid clinical trials. It is therefore of note that even within such a “pure” group, the presence of white matter microangiopathy was noted on MRI and predicted an “absence” of amyloid. Others have commented on white matter microangiopathy in AD radiologically (38). In our study, significant white matter microangiopathy was more frequent in Aβ− than in the Aβ+ subgroup and probably remains a major driving force behind cognitive decline in the Aβ− individuals. The difference is very close to statistical significance. This finding is in line with studies where patients were diagnosed from a dementia clinic based on clinical-radiological features. For instance, Jeong et al. (39) found no difference in the white matter burden between Alzheimer and non-Alzheimer groups statistically but a closer look at the data reveals that the Alzheimer group had more of minimal vascular disease while the other group had more of moderate vascular burden.

While microhemorrhages do constitute an important substrate for cognitive decline and dementia (40, 41), for the purpose of anti-Aβ trials, people with more than 5 microbleeds are generally excluded at the point of screening (42). Since our study cohort was recruited from the screening point of clinical trials unit, the patients did not demonstrate a significant occurrence of microhemorrhages that could be analyzed statistically. This, in general, would be applicable to clinicians screening patients in memory clinics worldwide, who are the primary addressee of this scoring system.

### 4.4 Clinical algorithm score

The Clinical Amyloid Positivity prediction Score or CAPS is proposed here as a simple clinical-radiological score that can be used to predict the probability of an individual with cognitive problems to be Aβ+ either on CSF or PET. This score considers three parameters: rapid cognitive decline (as determined by MMSE score); presence and severity of NPS (as determined by NPI-Q score), and white matter disease (as determined by Fazekas score on MRI). These tools are frequently used in any clinic dealing with individuals having memory or other cognitive complaints. In the present study, CAPS was able to classify more than 85% of Aβ+ cases correctly, while the predictive value for Aβ− individuals was slightly lower in the range of 80%. This simple clinical tool is easy to apply because the parameters involved here are quite accessible to clinicians. Any clinician following a patient with cognitive decline will be able to apply the CAPS scoring system and refer the patients for biomarker analysis accordingly. To our understanding, this is the first time a clinical scoring system to predict β-amyloid positivity has been developed.

### 4.5 Medial temporal lobe atrophy (CAPS-MT)

Medial temporal lobar atrophy is a well-known marker of neurodegeneration in AD (43). MTLA alone has limited reliability for predicting underlying Alzheimer pathology but becomes quite predictive when supplemented with cognitive scores and Apo E genotype (39). It has been found to be associated with progression of cognitive decline in Aβ+ individuals predicting conversion of MCI to dementia (44). In our analysis, we did not find any significant difference between the two subgroups (Aβ+. Aβ−) in terms of MTLA. Nevertheless, a greater degree of MTL atrophy in Aβ + individuals than their Aβ− counterparts was noted in our study cohort. If the MTLA scores are included alongside CAPS, as in CAPS-MT, the AUC improves from 0.867 to 0.900, suggesting a better discrimination than CAPS alone. That said, MTLA is known to be independently associated with rapid cognitive decline in Aβ + people and rapid decline is already a component of CAPS. Since CAPS is designed to be a simple clinical tool for predicting β-amyloid positivity in any setting, MTLA was not included in the component of CAPS, although inclusion of this variable can improve the discrimination. If adequate neuroradiological resources are available for assessment of MTL atrophy, CAPS-MT can prove to be strongly predictive of Aβ positivity status in an individual with “Alzheimer Syndrome.”

### 4.6 CAPS: context of use

It should be noted that almost half of our cohort was classified for their Aβ status based on their CSF result. This has around 90% sensitivity and specificity compared to Aβ PET scans (45), and brain PET remains a more direct method of detecting Aβ deposition. This understandably may have affected the interpretation of CAPS to a minor extent. That said even Aβ PET scans are not 100% sensitive or specific when compared to pathological examination of the brain (46). In fact, visual read of Aβ PET scans, an approved method for amyloid detection by the FDA, falls short of perfect concordance with quantitative Aβ PET measures by around 14% (47). Likewise, any developing biomarker that uses Aβ PET or CSF as a gold standard for comparisons would have at least some amount of embedded imperfection whereas comparison with neuropathological examination of the brain is not always practicable.

The reliability of CAPS did not reach 100% to predict Aβ+/Aβ− status in an individual. We found in our analysis that three out of 28 Aβ + persons did score below 3 on CAPS resulting in false negativity while four of 20 persons with Aβ− status scored 3 or more, indicating the presence of a false positive group.

A closer look at the three false negative cases reveals that two of them were over 80 years of age. In such older individuals, Aβ positivity is not uncommon, even without cognitive impairment (48), and the negative predictive value of CAPS may decline at this point. At this older age due to the presence of additional vascular lesions and Lewy bodies producing symptoms, cognitive impairment is often caused by a mixed effect of these pathologies complicating the picture further (48–50). Notably, both had a MTLA score of 4 which indicates that they were correctly classified according to CAPS-MT. The one other subject present in the false negative group was of age 69 years representing typical late onset AD. They had a score of 2 which is a borderline score, and they did not qualify on CAPS because of the absence of NPS. The MTL atrophy score in this individual was 1, reflecting minimal atrophy pattern and the fact that they would not have been correctly classified even when considering the magnitude of medial temporal atrophy. Not all people with clinical Alzheimer syndrome do present with notable NPS (28) or significant MTL atrophy (51) and these clinical situations call our attention to unusual features of some AD individuals, which may impact on their CAPS score.

The false positive group of CAPS (high CAPS score, but no evidence of amyloid on testing), included two individuals of age less than 60 years with early onset NPS. While NPS are clearly common in young onset AD with abnormal amyloid and tau pathology (52), it must be understood that on occasion, individuals with “Alzheimer Syndrome” due to non-amyloid pathology can also demonstrate early NPS, particularly in some younger individuals. The other two persons had a typical Alzheimer Disease clinical presentation, with CAPS score of 3 or 4. There were no particular features to distinguish them from the Aβ + individuals. A score of 4 has highest predictivity when using CAPS. Interestingly, 3/4 of these people had a MTLA score of 1, reflecting better classification accuracy of CAPS-MT. There can evidently still be some cases with clinical presentation of AD who cannot be distinguished clinically, and these will present a difficult clinical challenge.

The predictive values of CAPS (or CAPS-MT) depend to some extent on the anticipated prevalence of Aβ positivity in a particular age group, with 60–80 years having a clinical diagnosis of “Alzheimer Syndrome” being the group with highest predictivity. People above and below this age range may have higher incidences of false negativity and positivity, respectively on CAPS due to a higher and lower Aβ prevalence. Notably, a score of 4 was found to demonstrate highest positive predictivity whereas a score of 0 or 1 has the most negative predictive value. Inaccurate classification, when they occurred, were mostly associated with a score of 2 or 3 and outside the age range of 60–80 years. Accordingly, the scoring zone of 2 and 3 can be considered intermediate where more caution needs to be exercised particularly when the age of the individual is below 60 or above 80 years. More clinical, imaging and biomarker information are needed at this point to correctly stratify these candidates. One such parameter can be MTL atrophy which we observed has a higher accuracy when combined with CAPS. Similar situations are also encountered when working with fluid biomarkers in AD to the point that use of two age-related cut-offs are recommended by experts where the intermediate result group is followed up with a confirmatory test, such as PET, or repeat fluid biomarkers (46).

Clinical β-amyloid positivity prediction score holistically demonstrated a sensitivity close to 90% and specificity of 80% in distinguishing the Aβ+ and Aβ− subgroups. When combined with MTLA, these figures reach the 90% mark, which is the sensitivity/specificity for CSF based biomarkers. In no way, we are claiming that CAPS/CAPS-MT can replace biomarker assays. It is obvious that evidence of Aβ deposition in the brain is needed for someone to proceed for the anti-amyloid therapies. This scoring system is meant to help clinicians triage the patients who have a higher likelihood of turning out Aβ + on further confirmatory testing. This becomes meaningful in real-time clinical situations, given that the scoring system is based on simple clinical-radiological observation only and is meant to be followed by higher accuracy biomarker assays for AD. Most patients seen by general physicians and specialists can be accurately stratified in this method, which will aid in referral for anti-amyloid therapy.

### 4.7 Limitations

Our study has a few limitations, which are as follows: (1) the β-amyloid status was determined in most cases by using the CSF Aβ 42/40 ratio and in a few cases by PET. This can be attributed to the fact that the participants were selected from the memory clinic, where CSF analysis is a standard way to detect biomarkers for AD. A few patients went on to the stage of β-amyloid PET studies as part of their screening process for clinical trials. Therefore, the β-amyloid prediction is more biased toward CSF values. That said CSF and PET β-amyloid have a concordance of more than 90% in detecting AD pathology (45). (2) The sample size is relatively small. Given that this was only a single center study conducted within the time frame of only 1 year, the number of individuals with available β-amyloid biomarker status was limited. We are in the process of validating this scoring system in a larger national sample of individuals with dementia being made available in Canada through the Canadian Consortium on Neurodegeneration in Aging (CCNA). (3) Like every other biomarker, in some clinical situations, CAPS is not well applicable and needs caution before being employed. For instance, young onset dementia with early NPS may represent false positivity while age > 80 years may lead to false negative results due to reasons explained already. In addition, the sample we collected is based on a memory clinic where people do already have a diagnosis of clinical Alzheimer syndrome as per the NIA-AA criteria (32) before being referred to clinical trials screening. Anyone fulfilling the clinical criteria for Dementia with Lewy Bodies or other forms of dementia would, by default, have been excluded before referral. Therefore, the CAPS scheme holds true for clinical Alzheimer Syndrome but is not meant to be applied when the working diagnosis is already an alternative disease such as Lewy Body Disease or Frontotemporal dementia. It is meant to distinguish between Aβ+ and Aβ− negative subgroups with clinical Alzheimer syndrome or phenotype, exclusively.

## 5 Conclusion

Rapidly available clinical tools or algorithms for predicting amyloid positivity in individuals with clinical “Alzheimer Syndrome” have hitherto not been proposed. Current accurate biomarkers such as amyloid PET and even CSF evaluation are not actually available to the great majority of practicing physicians. CAPS is a simple clinical tool for use in primary care and memory clinic settings to predict Aβ positivity in individuals with clinical Alzheimer Syndrome. A score of 3 or 4 on CAPS has high predictability for β-amyloid positivity in Alzheimer Syndrome. We are currently in the process of validating CAPS in a larger cohort of people with cognitive impairment. Applying CAPS can help allocate limited resources for biomarkers in a more informed manner and thereby allow more people to have access to disease modifying therapies, specifically anti-β-amyloid medications, in AD.

## Data availability statement

The raw data supporting the conclusions of this article will be made available by the authors, without undue reservation.

## Ethics statement

The studies involving humans were approved by Baycrest Academy for Research and Education/Rotman Research Institute (REB#22-04). The studies were conducted in accordance with the local legislation and institutional requirements. Written informed consent for participation in this study was provided by the participants’ legal guardians/next of kin.

## Author contributions

DL: Conceptualization, Formal analysis, Writing – original draft, Writing – review & editing. BS-L: Formal analysis, Writing – original draft, Writing – review & editing. CR: Writing – original draft, Writing – review & editing. NV: Writing – original draft, Writing – review & editing. MF: Writing – original draft, Writing – review & editing. SA-S: Writing – original draft, Writing – review & editing. HC: Writing – original draft, Writing – review & editing, Conceptualization, Formal analysis, Funding acquisition, Methodology, Resources, Supervision.
